# Vestibular migraine

**DOI:** 10.1186/1129-2377-16-S1-A48

**Published:** 2015-09-28

**Authors:** Antonio Russo, Alessandro Tessitore, Gioacchino Tedeschi

**Affiliations:** Department of Medical, Surgical, Neurological, Metabolic and Aging Sciences, Second University of Naples, Naples, Italy; MRI Research Centre SUN-FISM, Second University of Naples, Naples, Italy; Institute for Diagnosis and Care “Hermitage Capodimonte”, Naples, Italy

Vestibular migraine (VM) has been increasingly recognized as a frequent cause of episodic vertigo, affecting up to 1% of the general population, with female preponderance[1].

Recently, both the Bárány Society and the Migraine Classification Subcommittee of the International Headache Society have proposed original diagnostic criteria for VM, which have been included in the recent edition of the International Classification of Headache Disorders (ICHD-3 beta version). VM diagnosis implies that vestibular symptoms are present during a migraine attack, with or without headache, in the absence of objectively demonstrated interictal vestibulopathy.

In the last decades, several studies have attempted to identify the electrophysiologic markers that could allow a distinction between VM and other vestibular disorders. Nevertheless, despite a growing body of literature, there is still an ongoing debate regarding whether VM origin is principally central or peripheral. However, during the past few years, the extensive application of advanced MRI techniques has contributed to significantly improving the understanding of VM pathophysiology. Functional and structural abnormalities have been detected in brain areas involved in multisensory vestibular control and central vestibular processing in patients with VM[2–4]. However, functional and structural alterations identified in patients experiencing VM also resemble those previously described for migraine. In conclusion, VM probably represents the pathophysiological paradigm of migraine and vestibular pathways connection.

Similarly to migraine pharmacological preventive therapy, VM treatment includes different prophylactic medications such as calcium channel blockers, beta-blockers, antiepileptic drugs and antidepressants, reporting consistent reduction of vertigo spells and or migraine attacks in a high rate of patients.
